# Have I Been Here? Sense of Location in People With Alzheimer's Disease

**DOI:** 10.3389/fnagi.2020.582525

**Published:** 2020-12-09

**Authors:** Ming-Chyi Pai, Shau-Shiun Jan

**Affiliations:** ^1^Division of Behavioral Neurology, Department of Neurology, College of Medicine, National Cheng Kung University Hospital, National Cheng Kung University, Tainan, Taiwan; ^2^Alzheimer's Disease Research Center, National Cheng Kung University Hospital, Tainan, Taiwan; ^3^Institute of Gerontology, College of Medicine, National Cheng Kung University, Tainan, Taiwan; ^4^Department of Aeronautics and Astronautics, National Cheng Kung University, Tainan, Taiwan

**Keywords:** dementia, Alzheimer's disease, spatial navigation, humans, sense of location

## Abstract

**Background:** When navigating in a particular space, a sense of being at a current location is of great help for the navigators in reaching their destination or getting back to the start. To accomplish this work, interwoven neural structures and neurons are called into play. This system is called the heading direction cell-place cell-grid cell circuit. Evidence from various neuroscience studies has revealed that the regions responsible for this circuit are damaged in the early stages of Alzheimer's disease (AD). This may explain why wayfinding difficulty is one of the most frequent symptoms in persons with AD. The aim of this study was to examine the sense of location (SoL) in persons with mild AD, persons with prodromal AD (prAD), and those who were cognitively unimpaired (CU).

**Methods:** We invited people with mild AD, prAD, and CU to participate in this study. The venue of the core experiment to assess SoL was a 660-m path located on the university campus. The participants were instructed to take a walk on the path and press a device to indicate their arrival at each of the five carefully chosen targets. The linear deviations from the target site were compared among the groups.

**Results:** A total of 20 AD, 28 prAD, and 29 CU persons completed the study. Their Mini-Mental State Examination scores were on average 20 (SD 3), 24 (SD 3), and 28 (SD 2). The groups were well differentiated regarding several measurements for cognitive ability and spatial navigation. As for the SoL, the hit rates of exact location with linear deviation of 16 m or less were 0.05, 0.54, and 0.86 for AD, prAD, and CU persons, respectively. The hit rates were well correlated with the presence of getting lost. Also, SoL differentiated well among CU, PrAD, and AD in terms of average linear deviation.

**Conclusions:** Our employing linear deviation by utilizing a grid-cell function device as an assessment for SoL showed distinct features among the three groups. This model can be used to develop more delicate devices or instruments to detect, monitor, and aid spatial navigation in persons with prAD and AD.

## Background

When moving in a physical space, a person may intermittently check the current location to assure being on the right path. This is called sense of location (SoL) (Jeffery, 2007). SoL helps an individual to reach his or her destination and return to the start point. To accomplish this seemingly simple work, interwoven neural structures are called into play.

It is known that a network in the brain provides navigators with knowledge of their current location and a representation of environmental scenes. This global positioning system (GPS)-like built-in neural network is called the place cell-heading direction cell-grid cell (PHG) system (Golob et al., 2001; Parron and Save, 2004; Hafting et al., 2005). The neural structures composing this system overlap with the regions which are damaged in the initial stage of Alzheimer's disease (AD) (Braak and Del Tredici, 2015), including the entorhinal cortex and its connected regions. This may explain why navigation impairment and getting lost (GL) are two of the incipient symptoms in persons with AD (Pai and Hsiao, 2002; Pai and Jacobs, 2004).

SoL, together with attention (Baddeley, 1986), landmark recognition (Lee and Pai, 2012), egocentric route following (Vogeley and Flink, 2003), forming and using cognitive maps (Jheng and Pai, 2009), translations between different spatial representations (Vann et al., 2009; Pai and Yang, 2013), and decision making (Janzen and van Turennout, 2004), plays a critical role in our daily navigation. Over the past decades, studies focusing on human navigation abilities have been carried out on participants, including cognitively unimpaired (CU) individuals, people living with AD, or people with mild cognitive impairment (MCI) (Hort et al., 2007; Gazova et al., 2012; Lithfous et al., 2013; Lester et al., 2017; Coughlan et al., 2018; Zanco et al., 2018). The mechanisms for spatial navigation impairment (SNI) in early AD, however, are not well understood.

Given that SNI is such an important issue in dementia care, learning more about the mechanism is helpful for creating techniques to prevent GL events (Pai and Lee, 2016). The aim of this study was to examine SoL in persons living with early-clinical-stage AD.

## Methods

### Participants

We invited people with mild AD and those with prodromal AD (prAD) from a dementia special clinic and the CU people from the community at large to join this study. A diagnosis of AD was made according to the criteria developed by the National Institute on Aging–Alzheimer's Association workgroups on diagnostic guidelines for AD (McKhann et al., 2011). For the AD group, only those with a Clinical Dementia Rating (CDR) Scale score of 1.0 were included. The clinical criteria for the participants with prAD were as follows: (1) subjective memory complaints confirmed by family members, (2) Mini-Mental State Examination scores between 24 and 30 or equivalently adjusted for educational level, (3) objective memory impairment for age, (4) a CDR of 0.5, (5) largely intact functional activities of daily living, and (6) absence of dementia (Petersen et al., 1999; Busse et al., 2003; Petersen, 2004; Winblad et al., 2004; Suk and Shen, 2014). A diagnosis of AD or prAD was supported by medial temporal atrophy and/or posterior cortical atrophy *via* brain MRI and perfusion deficit in either the precuneus and/or the posterior parietal lobes and/or the posterior cingulate gyrus *via* brain SPECT (Scheltens et al., 1992; Matsuda, 2007; Ramusino et al., 2019). The CU group were those with normal mental states and who lived completely independently. These were mostly family members, especially spouses of patients.

### Basic Neuropsychological Tests

We administered the Cognitive Abilities Screening Instrument (CASI) (Teng et al., 1994) and the Neuropsychological Batteries developed by the Consortium to Establish a Registration of Alzheimer's Disease (CERAD) (Morris et al., 1989) to examine general and specific cognitive functions. The Questionnaire on Everyday Navigational Ability (QuENA) (Pai et al., 2012) was used to assess the participants' navigational abilities and behavior. Moreover, all participants were assessed for their abilities of perception of time and distance (Bindra and Waksberg, 1956; Grondin, 2010; Bian and Anderson, 2013), in which both perceptions were measured with respect to verbal estimation and production.

### Core Experiments

**Figure 1:**
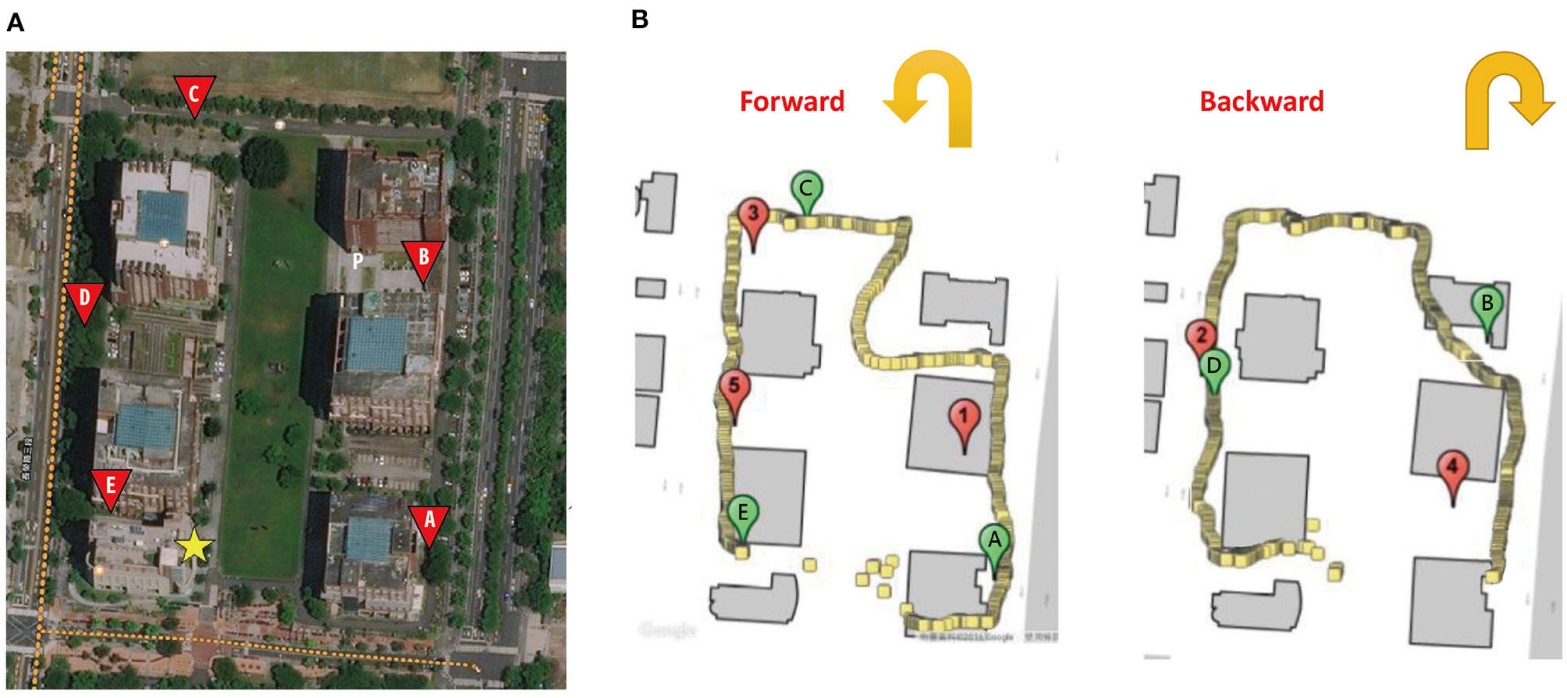
**(A)** The aerial photo of the campus. **(B)** The path, target site locations (marked with letters A–E), the participant trace recorded by Pai-Jan device, and the areas that the participant reached (marked with numbers 1–5). The yellow star indicates the starting point of the path. Letter P indicates the participant.

The core experiment was carried out on Tzu-Chiang Campus at National Cheng Kung University. Each participant was shown a map illustrating the layout of the campus throughout the study, in which several rectangle turns are on the path for the participants to walk. The length of the path was 660 m, on which five sites (A–E) were carefully chosen to prevent individuals from seeing the starting point. The participants, prior to the experiment, were instructed to approach the five target sites one after another and touch the screen of a tablet personal computer (PC) with an innovatively designed Pai-Jan (PJ) device (Pai et al., 2016) upon judging that they had reached the target. Figure 1A provides an aerial photo of the campus and the PJ device interface on which the designed path and the location of the target sites were shown. The location of the participant could also be seen on the PJ device interface.

Accompanied by researchers, the participants took a forward (with targets A, C, and E) and a reverse route (with targets B and D). The sequence of forward and reverse routes was counterbalanced among the participants to eliminate the effect produced by the two directions. The PJ device recorded the geographical location of the point by GPS when a participant touched the screen. The linear deviation of the participant from the target at each site could be precisely calculated and was treated as a variable to be compared among the three groups. Figure 1B provides the trace of a participant as recorded on the PJ device. Moreover, at each target, the PJ device also showed an arrow for the participants to point in the direction of the origin. The angular deviation from the correct direction was compared as a function of path integration (Allan, 1979; Howett et al., 2019). The design of the core experiment was akin to a daily activity when one goes outside to reach a destination held in mind, and it was an ecologically valid setting to reveal real-world evidence.

### Institutional Review Board

All procedures were approved by the National Cheng Kung University Hospital Institutional Review Board for the Protection of Human Subjects. All the participants provided informed consent before participating in this study.

### Data Analyses

Descriptive statistics were presented as means ± standard deviation. SPSS (version 22.0) for Windows was used for statistical analysis. Baseline demographic characteristics, including age, education years, CASI scores, age at onset, and CDR scores, were coded as continuous variables. Other demographic characteristics, such as gender, were coded as category variables. One-way analysis of variance or Kruskal—Wallis test was used to analyze the demographic factors, and analysis of covariance was applied to analyze CASI, CERAD, time and distance perception, vector to the start, SoL, and the QuENA. *Post hoc* analysis, with the Bonferroni test, was used to compare the differences between each group. Pearson correlation analysis was used to check the correlations between the performance of the SoL and that of the QuENA. All the statistical tests were two-tailed, and significance levels were set at a *p*-value of < 0.05.

## Results

### Basic Data

A total of 77 subjects completed the study. Table 1 presents the demographic variables in the three groups: CU, PrAD, and AD subjects.

**Table 1:** Demographic and neuropsychological tests.

	**CU**	**PrAD**	**AD**	***p*-value**	***Post hoc***
	**(*n* = 29)**	**(*n* = 28)**	**(*n* = 20)**		
Gender (M:F)^g^	14:15	19:9	7:13	0.071	
Age (years)^h^	69.5 (6.7)	71.0 (7.7)	73.5 (8.4)	0.192	
Education (years) ^i^	11.9 (3.7)	10.4 (3.8)	8.8 (3.3)	0.019^*^	a^**^
MMSE^i^	27.5 (2.2)	24.1 (3.4)	20.2 (2.9)	0.000^***^	a^***^, b^***^, c^***^
CDR (SoB)^i^	NA	1.2 (0.7)	4.1 (1.2)	0.000^***^	e^***^
Age at onset (y ears)^h^	NA	65.8 (9.7)	68.7 (6.6)	0.288	
**CASI**^**j**^					
Remote memory	10.0 (0.2)	10.0 (0.0)	9.9 (0.5)	0.250	
Recent memory	10.6 (1.1)	7.0 (3.2)	3.3 (2.7)	0.000^***^	a^***^, b^***^, c^***^
Attention	7.5 (0.7)	7.0 (1.3)	7.1 (0.9)	0.379	
Mental manipulation	9.0 (1.3)	7.9 (2.5)	7.8 (2.5)	0.332	
Orientation	17.8 (0.6)	16.9 (1.1)	10.8 (4.1)	0.000^***^	a^***^, b^***^
Abstract thinking	9.6 (1.7)	7.5 (1.6)	7.5 (1.5)	0.000^***^	a^**^, c^***^
Language	9.7 (0.7)	9.4 (1.0)	9.3 (0.9)	0.567	
Drawing	9.9 (0.3)	8.9 (2.2)	9.3 (1.1)	0.063	
Animal	9.0 (1.9)	7.6 (2.4)	6.4 (2.4)	0.009^**^	a^**^
CASI total score	92.6 (5.8)	82.2 (9.8)	71.1 (6.9)	0.000^***^	a^***^, b^***^, c^***^
**CERAD**^**j**^					
Verbal fluency	14.9 (3.5)	12.5 (3.7)	10.7 (2.2)	0.000^***^	a^**^, c^*^
Boston naming test	14.7 (1.0)	14.2 (1.0)	13.7 (1.5)	0.045^*^	a^*^
Word list memory	21.7 (3.3)	17.1 (3.7)	13.9 (2.4)	0.000^***^	a^***^, b^*^, c^***^
Constructional praxis	10.4 (0.9)	10.0 (1.2)	10.4 (1.5)	0.211	
Word list recall	7.7 (1.6)	4.5 (2.0)	1.8 (1.8)	0.000^***^	a^***^, b^***^, c^***^
Word list recognition	18.8 (1.7)	16.8 (2.3)	12.9 (2.4)	0.000^***^	a^***^, b^***^, c^**^
Recall constructional praxis	8.9 (2.3)	5.1 (3.8)	1.7 (2.5)	0.000^***^	a^***^, b^**^, c^***^
**Trail making test (TMT)**^**g**^					
TMTa (*N*; 0/1~5/>5)	29; 26/2/1	28; 22/4/2	19; 13/5/1	0.402	
TMTb (*N*; 0/1~5/>5)	29; 14/11/4	27; 8/11/8	18; 1/6/11	0.003^**^	a^**^
TMTc (*N*; 0/1~5/>5)	29; 27/2/0	28; 24/3/1	19; 11/6/2	0.034^*^	a^**^
**QuENA**^**j**^					
Landmark and scene agnosia	1.2 (1.4)	2.8 (1.6)	3.2 (1.8)	0.000^***^	d^***^, f^**^
Egocentric agnosia	0.7 (0.9)	1.9 (1.0)	2.5 (1.1)	0.000^***^	d^***^, f^***^
Inattention	1.1 (0.8)	2.1 (1.4)	2.3 (1.8)	0.007^**^	d^*^, f^*^
Heading disorientation	0.9 (1.0)	2.1 (1.7)	2.4 (1.5)	0.001^***^	d^**^, f^**^
QuENA total score	4.0 (3.3)	8.5 (5.0)	10.3 (4.3)	0.000^***^	d^***^, f^***^
Getting lost, *N* (%)^g^	NA	9 (32.1%)	10 (50.0%)	0.260	
**Magnetic resonance imaging**^**h**^					
MTA visual rating scale [*N*; M (SD)]	NA	27; 1.7 (1.0)	19; 2.6 (1.3)	0.019^*^	e^*^
PA visual rating scale [*N*; M (SD)]	NA	27; 1.2 (0.8)	19; 1.5 (1.0)	0.444	

*Data are presented as mean (standard deviation)*.

*CU, cognitively unimpaired; PrAD, prodromal AD; AD, Alzheimer's disease; a, CU > AD; b, PrAD > AD; c, CU > PrAD; d, CU < AD; e, PrAD < AD; f, CU < PrAD; g, chi-square test p-value; h, ANOVA p-value; i, Kruskal–Wallis test for comparison among groups and Dunn's test for post hoc; j, ANCOVA; MMSE, Mini-mental State Examination; CDR SoB, Clinical Dementia Rating Sum of Box; CASI, Cognitive Abilities Screening Instrument; CERAD, Consortium to Establish a Registry for Alzheimer's Disease, post hoc analysis by Scheffe Test; QuENA, Questionnaire on Everyday Navigational; MTA, medial temporal lobe atrophy; PA, posterior atrophy; in visual rating scale: M, mean; SD, standard deviation*.

* *p ≤ 0.05*,

** *p ≤ 0.01*,

*** *p ≤ 0.001*.

As shown in Table 1, the CASI and the CERAD scores revealed significant between-group differences. AD and prAD were more impaired in spatial navigation than CU as assessed by QuENA, while they showed no difference between each other. This finding was compatible with the hypothesis that the neural structures related to spatial navigation were damaged in the clinical stage of prAD. A chi-square analysis in GL percentage also revealed significant between-group distribution differences. It was also noted that education year differed with statistical significance between CU and AD people.

Regarding time perception, comparing prAD and AD patients with the CU group, verbal time estimation deviation and time production deviation showed significant differences (Table 2). AD produced more deviation in the test of distance production than prAD did; otherwise, no difference was detected among the groups in assessing perception of distance.

**Table 2:** Time perception and self-motion deviation.

	**CU (*n* = 25)**	**CU (*n* = 26)**	**PrAD (*n* = 25)**	**PrAD (*n* = 28)**	**AD (*n* = 18)**	**AD (*n* = 15)**	***p*-value**	***Post hoc***
**Verbal time estimation deviation (second)**								
10	3.7 (4.1)		4.1 (3.7)		4.7 (3.6)		0.707	
30	6.0 (7.2)		9.2 (7.1)		12.7 (9.7)		0.029^*^	A
60	12.1 (12.8)		17.6 (15.7)		24.8 (14.9)		0.022^*^	A
**Time production deviation (second)**								
10	2.0 (1.3)		2.8 (1.9)		2.8 (2.0)		0.139	
30	6.1 (5.3)		8.6 (6.0)		9.7 (6.2)		0.116	
60	11.0 (10.3)		17.5 (11.2)		22.1 (10.5)		0.005^**^	A
**Verbal distance estimation deviation (meter)**								
10		2.4 (1.9)		2.4 (2.0)		3.3 (2.7)	0.361	
50		12.1 (8.8)		14.2 (10.9)		18.1 (9.1)	0.171	
100		30.6 (21.7)		29.9 (28.9)		42.1 (30.4)	0.321	
**Distance production deviation (meter)**								
10	4.2 (3.5)			5.4 (6.1)		4.3 (3.7)	0.581	
50	19.3 (14.8)			12.7 (9.6)		17.0 (11.1)	0.135	
100	34.9 (28.2)			22.8 (16.9)		42.8 (19.3)	0.017^*^	B

*Data are presented as mean (standard deviation). Post hoc analysis by Dunn's test*.

*CU, cognitively unimpaired; PrAD, prodromal AD; AD, Alzheimer's disease; a, CU < AD; b, PrAD < AD; c, CU < PrAD*.

* *p ≤ 0.05*,

** *p ≤ 0.01; outliers (>2.5 standard deviation) are excluded*.

### Core Experiments

**Figure 2:**
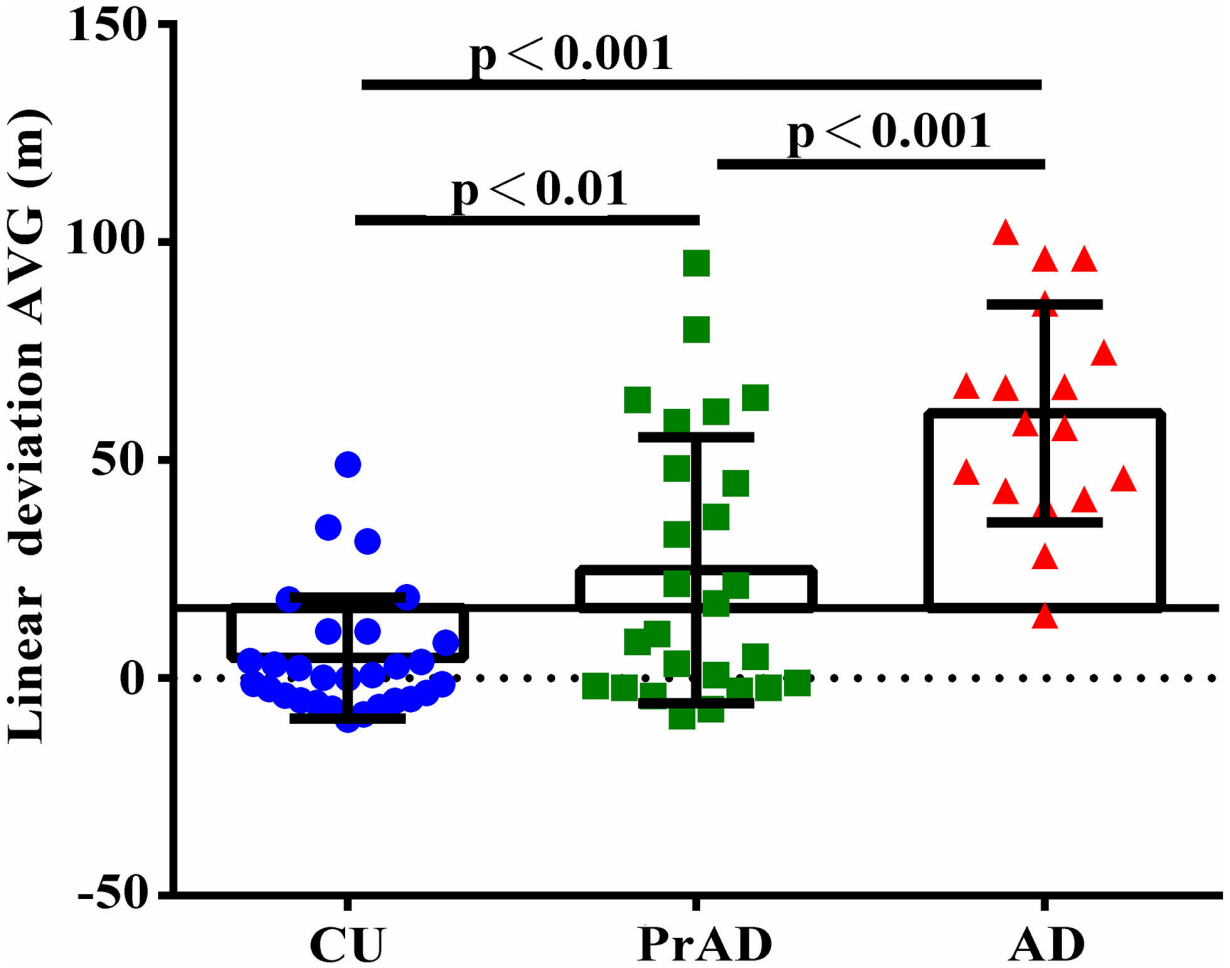
The comparison of linear deviation among groups with *post hoc* comparison.

As shown in Table 3, the SoL clearly differentiated among CU, prAD, and AD. For example, the average linear deviation in meters in the CU, prAD, and AD were 19 ± 13, 39 ± 31, and 79 ± 25, respectively (*p* < 0.001, *post hoc* comparisons AD vs. prAD: *p* < 0.001; AD vs. CU: *p* < 0.001; prAD vs. CU: *p* < 0.01). Figure 2 illustrates the scattered plot of individual linear deviation together with the *post hoc* comparisons among CU, prAD, and AD. A difference was detected between AD and both prAD and CU in vector to the start in degrees, while no difference was noted between prAD and CU. Thus, the results showed that vector to the start might not serve as significant predictors for dementia status at the follow-up stage, particularly during the early stages of the disease.

**Table 3:** Sense of location.

	**CU (*n* = 29)**	**PrAD (*n* = 28)**	**AD (*n* = 20)**	***p*-value**	***Post hoc***
**Linear deviation (meter)**					
P1	19.5 (32.3)	39.0 (46.2)	90.5 (62.0)	0.000^***^	a^***^, b^***^
P2	23.5 (20.6)	35.4 (40.6)	59.6 (42.2)	0.029^*^	a^*^
P3	21.8 (27.6)	54.1 (62.5)	77.4 (59.0)	0.010^**^	a^**^
P4	15.5 (8.5)	37.7 (36.9)	88.4 (38.1)	0.000^***^	a^***^, b^***^, c^*^
P5	16.7 (13.8)	26.4 (21.9)	79.4 (62.7)	0.000^***^	a^***^, b^***^
AVG	19.4 (13.0)	38.5 (30.5)	79.3 (24.7)	0.000^***^	a^***^, b^***^, c^*^
**Vector to the start (degree)**					
P1	31.3 (34.2)	38.9 (40.8)	57.2 (45.7)	0.084	
P2	34.4 (23.4)	28.6 (22.0)	40.8 (25.2)	0.106	
P3	27.0 (33.8)	31.6 (27.9)	36.5 (39.4)	0.705	
P4	29.9 (26.2)	24.7 (20.9)	52.5 (49.1)	0.023^*^	b^*^
P5	34.1 (28.3)	45.1 (39.6)	71.9 (46.0)	0.021^*^	a^*^
AVG	31.3 (20.4)	33.8 (14.4)	51.8 (24.7)	0.004^**^	a^**^, b^*^

*Data are presented as mean (standard deviation). Post hoc analysis by ANCOVA*.

*AD, Alzheimer's disease; PrAD, prodromal AD; CU, cognitively unimpaired; a, CU < AD; b, PrAD < AD; c, CU < PrAD; AVG, average*.

* *p ≤ 0.05*,

** *p ≤ 0.01*,

*** *p ≤ 0.001*.

**Figure 3:**
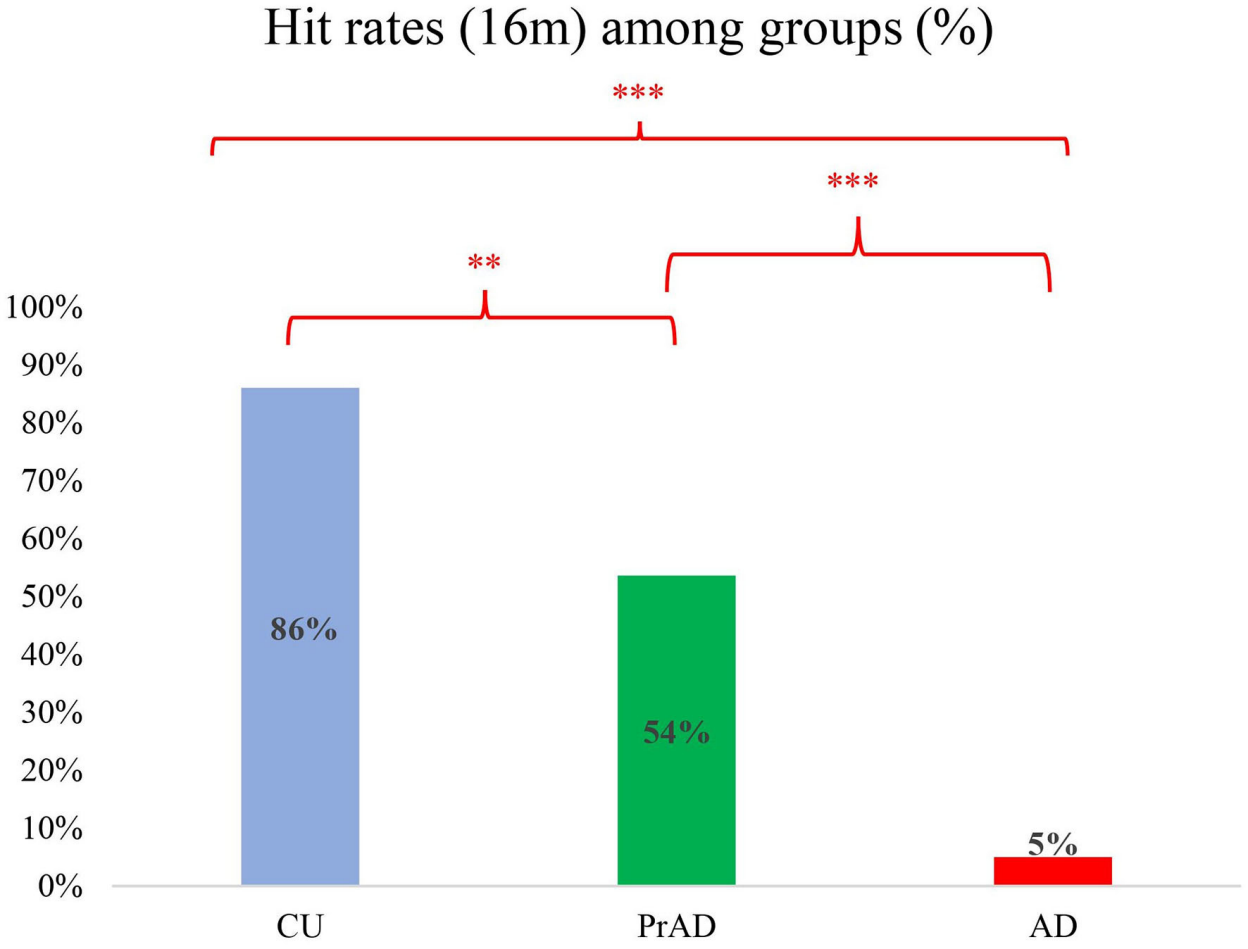
The hit rates among groups with *post hoc* comparison (**p < 0.01; ***p < 0.001).

Regarding linear deviation as a reference for diagnostic power, a deviation of 16 m was set as cutoff for comparison (Pai et al., 2016). The hit rates were 0.86, 0.54, and 0.05 for CU, prAD, and AD, respectively (*p* < 0.001, *post hoc* comparisons AD vs. prAD, *p* < 0.001; AD vs. CU, *p* < 0.001; prAD vs. CU, *p* < 0.01; Figure 3). The hit rates were well correlated with the presence of GL (correlation = 0.43 with *p*-value = 0.001).

The correlation between QuENA and linear deviation reached a statistical significance in average inattention for prAD patients as well as in average landmark scene agnosia and egocentric agnosia when considering all subjects (Table 4). Statistical significance was also attained in the correlation between the presence of GL and average landmark scene agnosia when prAD and AD subjects were considered (Table 5). When taking consideration of SoL and QuENA, a statistical significance was observed in several domains, including linear deviation with inattention for prAD subjects, and with landmark scene agnosia and with egocentric agnosia if all subjects were under consideration (Table 6).

**Table 4:** Pearson correlation between QuENA and linear deviation.

	**Landmark scene agnosia**	**Egocentric agnosia**	**Inattention**	**Heading disorientation**	**Total score**
**CU (*****n*** **=** **29)**					
A	−0.009	−0.083	−0.103	0.079	−0.027
B	−0.039	−0.147	−0.162	−0.196	−0.154
C	0.181	−0.278	−0.096	−0.106	−0.053
D	−0.108	0.052	−0.107	0.243	0.014
E	0.153	−0.249	−0.137	−0.082	−0.060
AVG	0.079	−0.253	−0.187	−0.053	−0.096
**PrAD (*****n*** **=** **27)^a^**					
A	−0.003	−0.171	−0.298	−0.250	−0.227
B	−0.287	−0.394^*^	−0.357	−0.111	−0.334
C	0.100	−0.216	−0.335	−0.099	−0.108
D	−0.201	−0.343	−0.410^*^	−0.161	−0.267
E	−0.285	−0.228	−0.115	−0.004	−0.124
AVG	−0.125	−0.360	−0.438^*^	−0.185	−0.285
**AD (*****n*** **=** **20)**					
A	−0.090	−0.088	0.360	0.016	0.093
B	0.117	0.180	−0.168	−0.091	−0.008
C	−0.017	0.063	0.089	−0.146	−0.006
D	−0.110	0.011	−0.433	−0.222	−0.297
E	−0.018	−0.153	−0.132	0.063	−0.076
AVG	−0.074	−0.033	−0.038	−0.136	−0.102
**Total (*****n*** **=** **76)**					
A	0.175	0.202	0.182	0.112	0.193
B	0.083	0.105	−0.097	0.038	0.031
C	0.263^*^	0.145	0.029	0.077	0.168
D	0.192	0.297^**^	−0.042	0.152	0.186
E	0.171	0.191	0.070	0.199	0.199
AVG	0.247^*^	0.261^*^	0.055	0.158	0.221

a *Missing data in this value*.

*CU, cognitively unimpaired; PrAD, prodromal AD; AD, Alzheimer's disease; AVG, average*.

* *p ≤ 0.05*,

** *p ≤ 0.01*.

**Table 5:** Pearson correlation between GL and QuENA and between GL and SoL.

	**QuENA**					**Sense of location**	
	**Landmark scene agnosia**	**Egocentric agnosia**	**Inattention**	**Heading disorientation**	**Total score**	**Liner deviation**	**Vector to the start**
PrAD (*n* = 28)	−0.247	−0.256	−0.024	−0.246	−0.117	−0.29	−0.00
AD (*n* = 20)	−0.431	−0.049	−0.176	−0.067	−0.285	0.04	0.11
PrAD + AD (*n* = 48)	−0.348^*^	−0.211	−0.112	−0.184	−0.210	−0.23	−0.03

*PrAD, prodromal AD; AD, Alzheimer's disease*.

* *p ≤ 0.05*.

**Table 6:** Pearson correlation between sense of location and QuENA.

	**Landmark scene agnosia**	**Egocentric agnosia**	**Inattention**	**Heading disorientation**	**Total score**
**CU (*****n*** **=** **29)**					
Linear deviation	0.079	−0.253	−0.187	−0.053	−0.096
Vector to the start	−0.003	−0.171	−0.023	0.035	−0.042
**PrAD (*****n*** **=** **28)**					
Linear deviation	−0.125	−0.360	−0.438^*^	−0.185	−0.285
Vector to the start	−0.139	−0.196	−0.174	0.005	−0.040
**AD (*****n*** **=** **20)**					
Linear deviation	−0.074	−0.033	−0.038	−0.136	−0.102
Vector to the start	−0.077	−0.134	−0.034	−0.435	−0.233
**PrAD** **+** **AD (*****n*** **=** **48)**					
Linear deviation	−0.019	−0.021	−0.171	−0.084	−0.067
Vector to the start	−0.045	−0.021	−0.049	−0.160	−0.036
**Total (*****n*** **=** **77)**					
Linear deviation	0.247^*^	0.261^*^	0.055	0.158	0.221
Vector to the start	0.086	0.080	0.049	0.005	0.085

*CU, cognitively unimpaired; PrAD, prodromal AD; AD, Alzheimer's disease*.

* *p ≤ 0.05*.

## Discussion

Traditionally, the strategies a person may adopt for everyday navigation can be divided to egocentric and allocentric with adequate landmark recognition (Lester et al., 2017; Schoberl et al., 2020a). In this study, the SoL measured egocentric and allocentric strategies as well as the translation between these two spatial representations (Pai and Yang, 2013), matching and judging the current location with reference to cognitive maps, and prefrontal functions (Jeffery, 2007). Hence, SoL reflected a more holistic approach to real-world navigation, and impairment of it may contribute to the occurrence of GL (Yatawara et al., 2017) and result in the inability to lead an independent life. Conceptually, SoL is analogous to the function of grid cells (Howett et al., 2019), and that the entorhinal cortex underpins navigation in other mammalian species is supported by the demonstration of entorhinal cortex grid cells in rats (Hafting et al., 2005), bats (Yartsev et al., 2011), monkeys (Killian and Buffalo, 2018), and humans (Jacobs et al., 2013). Since the entorhinal cortex and its connections are highly related to early pathological changes in AD patients, a device designed based on grid-cell function might be a good way to depict the ways in which how SNI troubles community-residing people with AD (Pai and Jacobs, 2004).

This study demonstrated that SoL, as assessed by linear deviation by a grid-cell function driven device, was more impaired in AD and PrAD than in CU. This distinct feature among the three groups was further supported by the hit rates of 16 m in terms of positioning precision (Figure 3). How one senses the current location in a specific surrounding is an interesting topic for neuroscientists. SoL helps navigators to judge one's present location relative to the start point or to the destination. As mentioned, to know if an individual has reached a pre-set destination in an environment, the individual may have to use multiple mental resources, including path integration, cognitive maps, landmark recognition, and translation of different spatial representations (Jheng and Pai, 2009; Lee and Pai, 2012; Pai and Yang, 2013). When applying this scenario to a person with PrAD or mild AD in a real-world situation who is ambulating on a daily route, it is reasonable to postulate that an impaired SoL may emerge intermittently long before the occurrence of his or her first-ever GL event because the individual may have impairment in one or more of the mentioned mental resources (Tu and Pai, 2006).

The performance of SoL observed in prAD in this study was in accordance with previous human navigation research (Howett et al., 2019). Although detailed history could not be obtained from these patients because of their impaired episodic memory, from clinical observation, many of them had spent extra time trying to recover the ability. In the current study, the neural substrates accountable for SoL function in both prAD and AD were supposedly damaged (Braak and Del Tredici, 2015), and the deficits of the SoL function shown in this study, both prAD and AD being worse than CU, supported this hypothesis.

From the concept of dead reckoning, the perception of time, distance or speed, and direction is critical for ocean sailors to reach a destination where no landmarks such as islets are present between the start point and the destination. In this study, we assessed perception of time and distance because the SoL depended on these basic abilities, and we tried to eliminate these confounders. Unexpectedly, the results revealed differences in several situations among the groups. How these differences would affect the SoL needs future well-designed research to answer.

The relationships among SoL, spatial navigation, and cognitive functions deserve further discussion. Except for moderate cognitive decline as in the clinical stage of mild and more advanced dementia, the cognitive functions and daily navigation abilities are not parallel. As mentioned, the cerebral cortical areas responsible for spatial navigation were supposedly damaged in prAD and mild AD, while a trend was present for a lower rate of GL in prAD to AD, though not reaching statistical significance. One reason was that factors unrelated to spatial cognition might prevent individuals with cognitive impairment from GL. For example, in their daily lives, people with prAD may preserve an adequate problem-solving ability and adopt compensatory strategies, such as using cues or indices as they are encountered with an impaired SoL. They may also call family members, ask others nearby or police officers for guidance, or take a taxi home. In this study, that the prAD had better performance on many items in CASI and in CERAD supports this hypothesis. Theoretically speaking, the parietal lobe as an integration center for multi-modality sensory information for successful navigation is functioning much better in prAD than in mild AD (Jeffery, 2007; Braak and Del Tredici, 2015). This is another explanation for the lower GL rate in prAD. On the other hand, that an impaired SoL in people at risk can be compensated for by proper means to prevent GL is insightful for comprehensive dementia care since a certain proportion of older adults suffer from cognitive impairment and are in danger of an unexpected GL event. In this study, that the AD and prAD groups manifested worse SoL and daily navigation abilities than the CU was compatible with the trend of the Alzheimer continuum in cognitive decline (Table 2). However, this finding was not in line with the daily navigation abilities in that the QuENA showed no difference between prAD and AD. From our previous study, the QuENA, as assessed by the caregivers or family members, did not faithfully reflect the awareness of the targeted persons with AD (Pai and Lee, 2016). The QuENA reported by prAD, as in this study, was usually underestimated in part due to preserved insight and anxiety and might lead to a discrepancy between the QuENA and the performance of SoL.

Regarding the vector to the start as a function of path integration, AD was worse than both prAD and CU, but no difference was detected between prAD and CU. Given that prAD's brain substrate responsible for path integration was damaged, however, the results did not support this hypothesis. In fact, the location of the neural substrates responsible for path integration in humans is controversial (Dudchenko, 2010). The path integration paradigm used in this study might be too simple, or other neural substrates may play a role in path integration as well. From these findings, it is suggested that SoL is better than path integration in differentiating CU, prAD, and mild AD.

Recently, papers focusing on spatial navigation as a marker to detect preclinical AD or to predict the conversion from MCI to AD have provided insightful information (Howett et al., 2019; Levine et al., 2020; Schoberl et al., 2020b). Our current results added further support for this possibility. Since the entorhinal cortex which contain lots of grid cells plays a sentinel role for signaling AD, how to crystalize the concept of grid-cell-related functions and create devices to differentiate prAD from other subtypes of MCI is mandatory (Moodley et al., 2015; Allison et al., 2016; Coughlan et al., 2018; Ritchie et al., 2018). These devices may also increase the confidence of physicians to make diagnoses of prAD and provide both patients and caregivers with information about the future progression. From our study, the presence of impaired SoL was linked to a danger of GL; hence, the caregivers can take action in advance to prevent the events.

Limitations included the relatively small number of AD participants. The design of the experiments in this study was complicated, which prevented more advanced AD patients from completing the procedures. Only cases with very mild or mild AD were feasible to join and complete the study. Another limitation concerns the lack of biomarkers, such as cerebrospinal fluid or amyloid positron emission tomography, to support a diagnosis of prAD or AD. However, this is a real-world difficulty; cheaper and more reliable biomarkers are needed to conquer this limitation.

Collectively, this work contributed to the growing body of evidence that impaired SoL appeared in the early clinical stage of AD and was associated with daily navigation impairment (Monacelli et al., 2003; Pai and Jacobs, 2004). Without efficient problem-solving strategies or help from pedestrians, an escort to the police station is inevitable; indeed a tragedy may even occur. Persistent and longitudinal studies of SoL in these participants are mandatory before its clinical application as a useful behavioral maker for AD.

## Data Availability Statement

The raw data supporting the conclusions of this article will be made available by the authors, without undue reservation.

## Ethics Statement

The studies involving human participants were reviewed and approved by National Cheng Kung University Hospital Institutional Review Board for the Protection of Human Subjects. The patients/participants provided their written informed consent to participate in this study.

## Author Contributions

M-CP undertook the literature search, study design, device design, conduct of data analysis, and editing of the author contributions and was mainly responsible for the revisions and drafts of the manuscript. S-SJ participated in the device design and data analysis and contributed to the revisions and the final draft of the manuscript. Both authors contributed to the article and approved the submitted version.

## Conflict of Interest

The authors declare that the research was conducted in the absence of any commercial or financial relationships that could be construed as a potential conflict of interest.
